# Neurofeedback for Management of Musculoskeletal Pain: A Randomized Control Trial Using DOMS

**DOI:** 10.1155/prm/8396291

**Published:** 2026-10-19

**Authors:** Katrine Aagaard Gregersen, Julie Brink Vilsøe, Felipe Rettore Andreis, Sabata Gervasio

**Affiliations:** ^1^ Department of Health Science and Technology Center for Neuroplasticity and Pain (CNAP) Aalborg University Aalborg Denmark aau.dk; ^2^ Department of Health Science and Technology Neural Engineering and Neurophysiology Aalborg University Aalborg Denmark aau.dk

## Abstract

**Introduction:**

Chronic musculoskeletal (MSK) pain is associated with maladaptive neuroplasticity, often reflected in altered alpha activity (8–13 Hz). Neurofeedback may offer a way to modulate this activity, yet most protocols target alpha activity during rest, despite movement being a common pain trigger. This study investigated whether neurofeedback during movement could modulate alpha activity and reduce pain in healthy individuals experiencing induced pain via delayed onset muscle soreness (DOMS).

**Method:**

This experiment was a randomized, two‐armed, parallel‐group trial. Twenty‐nine participants were included (15 females, mean age = 28 years). Exclusions included neurological, psychiatric, MSK conditions. Participants were randomly assigned to an intervention group (aiming to reduce alpha activity) or a control group (aiming to increase it). Alpha activity was assessed as the alpha power during rest and movement. DOMS was induced using eccentric exercise of muscle extensor carpi radialis in Session 1. Neurofeedback was administered in Session 2, where participants underwent three blocks of neurofeedback training, using individualized EEG‐based visual feedback to modulate alpha activity. Session 3 assessed potential lasting neurofeedback effects. Pain was rated across the three sessions, which took place 24–48 h apart.

**Results:**

DOMS induction was confirmed by increased pain (*p* < 0.001, *r* = 0.82) and reduced alpha activity during movement (*p* = 0.017, *r* = 0.49). Neurofeedback did not significantly alter alpha activity (*p* = 0.114, *W* = 0.05 for the control group, *p* = 0.338, *W* = 0.037 for the intervention group).

**Conclusion:**

A single neurofeedback session was insufficient to modulate brain activity or pain perception. Methodological factors, including limited training duration and participants’ strategies, may have contributed to the lack of an observable effect.

**Significance:**

Although neurofeedback did not result in significant changes after one session, the observed alpha modulation following DOMS highlights the relevance of targeting neuroplasticity during MSK pain. Future studies should optimize training protocols and consider multisession designs to enhance clinical applicability.

## 1. Introduction

WHO estimates that 20–33% of the world’s population is affected by chronic musculoskeletal (MSK) pain, making it a major cause of disability and reduced quality of life [1].

MSK pain often originates from repetitive movements or exertion, initially presenting as acute nociceptive pain. If unresolved, it might lead to maladaptive neuroplastic changes, including persistent activation of spinal neurons independent of peripheral stimuli and structural changes in the brain regions involved in pain processing [2, 3]. These changes can contribute to the development of pathological chronic pain [1, 4–6].

Current treatments include pharmacological and nonpharmacological approaches. Pharmacological treatments have side effects and can lead to dependence or addiction [7]. Nonpharmacological methods include passive modalities, such as heat or cold exposure, exercise, transcutaneous electrical nerve stimulation, together with management of psychological factors [1]. There is limited and varying evidence on the efficacy of passive interventions [1].

Exercise therapy is widely recommended as a treatment for chronic MSK pain and is supported by systematic reviews and international clinical guidelines [8, 9]. Nevertheless, treatment effects vary considerably between individuals, and long‐term adherence remains a challenge in clinical practice [9, 10]. Together, these limitations highlight the need to explore complementary approaches that may further improve treatment outcomes, such as neurofeedback.

Neurofeedback may counteract maladaptive plasticity by training individuals to regulate specific neural patterns [11, 12]. EEG‐based neurofeedback for management of pain typically targets frequency bands related to pain processing, where alpha activity has received particular attention due to its association with pain perception [7, 11, 13–15]. Several studies have reported pain reduction following protocols aimed at increasing alpha power during rest [13, 16], although other studies found no significant differences between active and sham groups, despite both showing increased alpha and reduced pain [18, 19]. These mixed findings suggest that evidence for a consistent association between alpha activity modulation and pain reduction remains inconclusive.

Importantly, MSK pain is often movement‐evoked, yet most neurofeedback protocols are designed for resting conditions. Mrachacz‐Kersting et al. [20] demonstrated that targeting alpha reduction during movement led to reductions in both alpha activity and pain after three sessions [20]. Similarly, Gervasio et al. [21] found that individuals with chronic MSK pain exhibited increased alpha power during movement but not at rest, compared to healthy controls, highlighting the relevance of movement‐specific alpha modulation [21].

This study aims to provide a proof of concept for the effectiveness on neurofeedback in modulating alpha activity during movement in the context of MSK pain management. Delayed onset muscle soreness (DOMS), which induces acute MSK pain 24–48 h after exercise, has been proposed as a suitable experimental model of chronic MSK pain [22]. Moreover, previous research has shown that a single neurofeedback session can effectively modulate event‐related alpha activity in healthy participants [23]. Therefore, the current study investigates how DOMS‐induced pain affects alpha activity during movement and whether a single neurofeedback session can modulate these changes in healthy individuals.

## 2. Method

### 2.1. Participants

This experiment was a randomized, two‐armed, parallel‐group trial designed to investigate the effects of EEG‐based neurofeedback on alpha activity and pain perception in healthy individuals following experimentally induced DOMS. CONSORT guidelines were followed for reporting. Participants were recruited via social media and oral outreach. A total of 30 individuals were enrolled. Inclusion criteria were age between 18 and 65 years and general good health, assessed based on participants’ self‐report [24]. Exclusion criteria included active drug use, pregnancy, or a previous or current diagnosis of neurological, psychiatric, or MSK disorders, recent surgery or fractures, participation in other clinical trials involving pain, or engaging in muscle training more than twice per week. Additional exclusion criteria were consumption of alcohol, caffeine, nicotine, or painkillers on test days, and inability to cooperate. One participant withdrew after the first session due to illness, resulting in 29 participants completing all three sessions. The final sample consisted of 15 females and 14 males (mean age = 28 years, SD = 9.6). The majority were right‐handed (*n* = 27, 93.1%).

Prior to participation, all individuals received written and verbal information about the study procedures, potential risks, and their rights as research participants. Written informed consent was obtained from all participants in accordance with the Declaration of Helsinki. The study was approved by the Scientific Ethical Committee of the North Denmark Region (N‐20240018).

### 2.2. Study Design

Participants were randomly assigned to either the intervention or control group in a 1:1 ratio using a computer‐generated randomization plan. Group allocation was blinded to participants.

Both groups received active neurofeedback based on their event‐related alpha activity. The intervention group was instructed to decrease alpha activity, while the control group was instructed to increase it. This design was chosen due to conflicting evidence regarding the directionality of alpha modulation in pain perception, allowing for exploratory analysis of its effect. It was hypothesized that both groups would be able to modulate alpha activity, but only the intervention group would experience a reduction in pain.

### 2.3. EEG and EMG Setup

EEG and EMG signals were recorded using the Enobio 8 system (Neuroelectrics, Spain). EEG electrodes were placed according to the international 10–10 System. The selected electrodes were Cz, with either C3 or C4 selected based on handedness, C3 for right‐handed and C4 for left‐handed participants.

EMG signals were recorded from *m*. Extensor carpi radialis. Electrode placement was determined by measuring the forearm length from the lateral epicondyle of the elbow to the radial and ulnar styloid processes at the wrist. The EMG electrode was positioned at 90% of this length, closest to the elbow [25]. The skin was shaved and disinfected prior to placement. Ambu Neuroline 720 electrodes with press‐stud connector were used. The reference electrode for both EEG and EMG was placed on the earlobe, with electrode gel applied to ensure signal quality.

### 2.4. Data Acquisition

Data were wirelessly transmitted from the Enobio 8 to the NIC2 software with a 500‐Hz sample rate. Custom interfaces developed in MATLAB App Designer (Mathworks Inc, USA) were used to receive and process data.

### 2.5. Data Processing

Signal processing was performed in MATLAB App Designer (Mathworks Inc, USA). EMG signals were filtered using a second‐order Butterworth high‐pass filter and band‐pass filer with cutoff frequencies of 55 Hz, and 2 and 40 Hz, respectively.

All data were reference to the earlobe. EEG data were visually inspected for quality during the recording. Recordings with extensive ocular and muscle artifacts were removed from analyzed data. Extensive artifacts were defined through offline visual inspection as artifact contamination that dominated the recording to such a degree that reliable interpretation of the EEG signal was not possible. This assessment was based on visual evaluation of the overall signal quality rather than an automated artifact rejection pipeline or a predefined quantitative criterion, allowing for individualized assessment of data quality on a recording‐by‐recording basis. However, as neurofeedback included real‐time processing of the data, this step was not implemented during the neurofeedback intervention.

EEG power was calculated using the fast Fourier transform (FFT), after which the power spectral density (PSD) was computed for the alpha band (8–12 Hz). PSD during movement was extracted using a single epoch from movement onset to one second postonset, with the 200–800‐ms window following movement onset identified as the interval of greatest alpha power increase [21]. For resting‐state EEG, the same epoch length was used with no overlap to ensure similar processing methods.

### 2.6. Experimental Protocol

The experiment consisted of three sessions. The overall structure of the experiment protocol can be seen in Figure 1.

**FIGURE 1:**
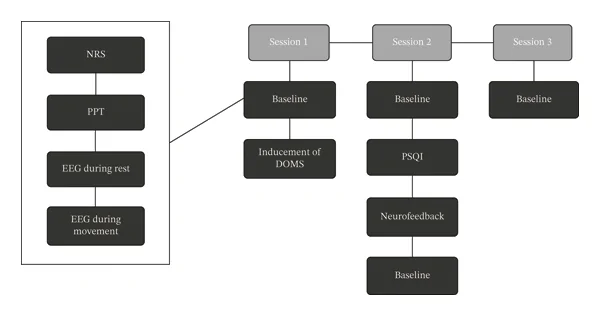
The experimental timeline and session structure. Session 1 includes a baseline and inducement of DOMS. Session 2 includes baseline, Pittsburgh Sleep Quality Index (PSQI), and a neurofeedback session. Session 3 is solely baseline. The baseline includes pressure pain threshold (PPT), numerical rating scale (NRS), EEG during rest, and EEG during movement.

Session 1 included a baseline measurement of alpha activity and pain perception followed by DOMS induction. Session 2, conducted 24–48 h later, included neurofeedback training across three blocks, with baseline measurements taken before and after training. Session 3, held 24–48 h after Session 2, included a final baseline measurement to assess lasting effects of neurofeedback. All sessions were conducted under controlled environmental conditions: lights were turned off, blinds, though not fully opaque, were lowered and background noise was minimized.

#### 2.6.1. Baseline

Participants were seated upright approximately 1 m from a fixed visual point at eye level, with arms resting on armrests. Baseline measurements included EEG recording during rest and movement, pressure pain threshold (PPT), numerical rating scale (NRS), and the Pittsburgh Sleep Quality Index (PSQI) collected in Session 2.

Resting EEG was recorded for 2 minutes with eyes open. Participants were instructed to minimize movement and focus on a visual point. Movement‐related EEG was recorded during five concentric movements of *m*. Extensor carpi radialis, each followed by a 5–10‐s relaxation. Prior to movement‐related EEG recording, a calibration procedure was performed to detect movement onset. A movement was detected when the average signal in a 50‐sample window exceeded a dynamic threshold, calculated from the mean and standard deviation of a previous 5000‐sample window, multiplied with a participant‐specific constant [26]. A beep signaled movement detection during calibration. If detection failed, the multiplier was adjusted and calibration repeated until consistent detection was achieved. The final multiplier was stored and used for movement detection during the session.

PPT was assessed using an Algometer type II (SBMEDIC), applying perpendicular pressure to the *m*. Extensor capri radialis. The pressure was applied at a point 1 cm distal to the EMG electrode. Participants pressed a handheld button when discomfort was felt. The procedure was repeated three times with 30 s between each procedure, and the mean was used as the final PPT. PPT assessments were performed by the same assessor (K. A. G), who was trained in the use of the algometer.

NRS was used to assess pain intensity during movement, where 0 indicated no pain and 10 indicated the worst imaginable pain.

#### 2.6.2. DOMS Protocol

DOMS was induced through repeated eccentric work of the *m*. Extensor carpi radialis, using a load equivalent to 80% of the participant’s one‐repetition maximum (1RM) [27]. The 1RM was determined by incrementally adjusting a handheld weight, starting at 8 kg, with 1‐kg increases or decreases based on performance. 1RM was defined as the highest weight where a participant would perform a full eccentric movement lasting 5 s. Participants rested for at least 30 s between attempts for 1RM. The current study employed a short rest period between attempts due to the small load increments minimizing cumulative fatigue effects.

To induce DOMS, eccentric movements were paced using a metronome set to 24 beats per minute, corresponding to one repetition every 5 seconds. The protocol consisted of three sets of 10 repetitions, with 2‐minute rest intervals.

The exercise was repeated 10 times, followed by a 2‐minute rest. If the participant was able to complete the set, the procedure with 10 repetitions and rest was repeated two additional times. If a participant was unable to complete a set, the weight was reduced by 1 kg, and the set was repeated [27].

#### 2.6.3. Neurofeedback

Neurofeedback training was conducted with participants seated in the same position as during baseline measurements. Participants performed self‐paced concentric contractions of *m*. Extensor carpi radialis approximately every 10 s. Each session included three blocks of training. The threshold for successful modulation was individualized based on the median event–related alpha power from the preceding EEG recording. For the intervention group (aiming to decrease alpha activity), the threshold was set to 80% of the median value; for the control group (aiming to increase alpha activity), it was set to 120%.

Participants were instructed to modulate their alpha activity to turn a visual feedback bar green (Figure 2).

**FIGURE 2:**
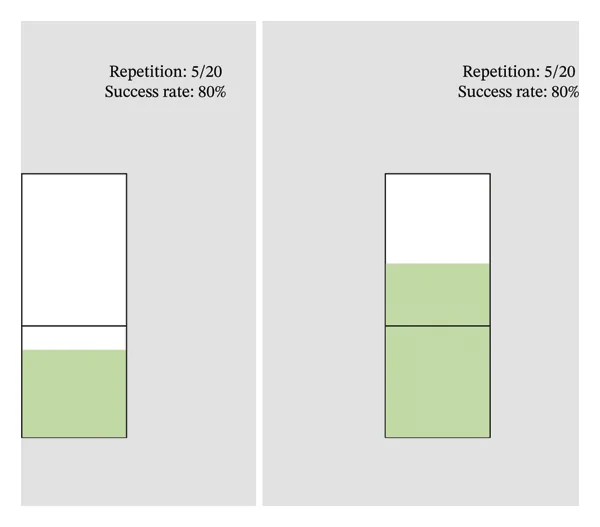
Interface used during the neurofeedback training. The bar represents the event‐related alpha activity. For the intervention group, successful modulation is achieved when the bar remains below the threshold line, turning green. For the control group, the bar must exceed the threshold line to turn green. The interface also displays the number of repetitions completed and the participant’s success rate.

For the intervention group, the bar had to remain below the threshold line; for the control group, it had to remain above. Each block consisted of 20 repetitions of eccentric contractions, followed by a 5‐minute break. Thresholds were recalculated after each block. No specific strategies were suggested to participants; however, postsession interviews were conducted to document self‐reported strategies, which were categorized based on participant descriptions and the framework by Autenrieth et al. [28].

### 2.7. Quality Assessment of Data

Poor electrode contact or extensive artifacts during EEG recordings were treated as missing data. Seven instances of low‐quality recordings were identified, with a maximum of two per variable. Missing values were handled using pairwise deletion. Variables with more than 10% of the missing data were excluded; however, no variables met this criterion.

### 2.8. Statistical Analysis

Data distribution was tested using a Shapiro–Wilk test, revealing that 73% of the variables were nonnormally distributed. Thereby, all statistical analyses were performed using nonparametric tests. Group comparisons (age, gender, hand dominance, alpha activity during movement and rest in Session 1, change in alpha activity after neurofeedback) were performed using the Mann–Whitney *U* test.

To evaluate the effectiveness of the DOMS protocol, changes in NRS, PPT, and alpha activity measurements during rest and movement between Session 1 and Session 2 (before neurofeedback) were compared using the Wilcoxon signed‐rank test.

For Mann–Whitney *U* and Wilcoxon signed‐rank tests, the effect size (*r*) was calculated as the absolute value of the standardized test statistic divided by the square root of the total sample size reflecting the magnitude of the difference between groups [3].

To analyze the alpha activity and how these changes between the sessions and the three blocks of neurofeedback z‐transformation will be used, *Z*‐transformation addresses the variability across participants, which may be influenced by differences in brain structure and function, head shape, and mental state.

The relationship between pain and sleep quality was assessed using Spearman’s rank‐order correlation between the change in NRS from Session 1 to Session 2 before neurofeedback and the PSQI scores. For correlations, Spearman’s rho was defined as the effect size.

To evaluate neurofeedback effect, Wilcoxon signed‐rank test was used to compare alpha activity before and after neurofeedback in Session 2. A Friedman test was used to assess alpha activity across blocks. Participants who reported strategies focused solely on movement pace or strength were excluded from some analysis to isolate cognitive modulation effects [29].

Successful neurofeedback modulation was defined as a ≥ 20% change in alpha activity compared to previous recordings. Based on Kropotov et al. [30], participants achieving this modulation in ≥ 60% of the training blocks were considered successful.

No data‐driven outlier removal was applied at either the epoch or participant level beyond predefined EEG artifact rejection criteria.

This study is reported in accordance with the CONSORT guidelines for randomized controlled trials.

## 3. Results

### 3.1. Comparison of Intervention and Control Groups, DOMS Effectiveness, and Sleep Quality

No significant statistical differences were found between the intervention and control groups in age, gender, dominant hand, or alpha during movement. A significant difference was observed in alpha activity during rest.

Values are shown in Table 1.

**TABLE 1:** Statistical comparisons between the control and intervention groups, including *p* values and the number of included participants for each test.

Comparison	Intervention group	Control group	Both groups	*p* value	Effect size (*r*)	Participants included
Alpha activity during movement session 1 (min–max)	−1.20925–1.93154	−1.29727–1.60271	−1.29727–1.94569	0.983	0.008	29
Alpha activity during rest session 1 (min–max)	−1.02243–2.30158	−1.02231–1.92449	−1.02243–2.30158	0.044	0.385	27

Significant increases in NRS and decreases in alpha activity during movement were observed between Session 1 and Session 2, indicating successful induction of DOMS. No significant changes were observed in PPT or resting alpha activity. All values are shown in Table 2.

**TABLE 2:** Statistical comparisons between Session 1 and Session 2 before neurofeedback, including *p* values and the number of included participants for each test.

Comparison	Value before DOMS	Value after DOMS	Change	*p* value	Effect size (*r*)	Participants included
NRS (*M* ± SD)	0.14 ± 0.351	1.86 ± 1.187	1.72 ± 1.162	< 0.001	0.82	29
PPT (*M* ± SD)	362.9 ± 224.312	336.38 ± 243.101	−26.52 ± 140.928	0.309	0.19	29
Alpha activity during movement (min–max)	−1.29727–1.94569	−0.98546–3.23354	−1.81115–1.83916	0.011	0.49	28
Alpha activity during rest (min–max)	−1.02243–2.30158	−1.12049–2.10564	−1.71283–2.37164	0.983	0.02	27

No significant relation was observed to the change in alpha activity during movement and change in NRS from baseline Session 1 to 2 (*p* = 0.816, *ρ* = −0.047), nor between NRS and alpha activity during movement in Session 2 (*p* = 0.886, *ρ* = −0.029).

No significant relation was observed between NRS and PSQI (*p* = 0.802, *ρ* = 0.049), indicating no relation between pain intensity and sleep quality.

### 3.2. Effectiveness of Neurofeedback

No significant changes in alpha activity were observed across blocks for each group (*n* = 12, *p* = 0.114, *W* = 0.05 for the control group, *n* = 12, *p* = 0.338, *W* = 0.037 for the intervention group). Furthermore, no significant between‐group difference was observed in the pre–post change in alpha activity (*n* = 24, *p* = 0.616, *r* = 0.10). Furthermore, as illustrated in Figure 3, both groups showed similar trends in alpha modulation over time, with overlapping standard deviations and no consistent group difference. Therefore, due to the lack of group‐specific effects, subsequent analyses were conducted on data of both groups combined to assess whether any changes could be seen from the neurofeedback independent of the group.

**FIGURE 3:**
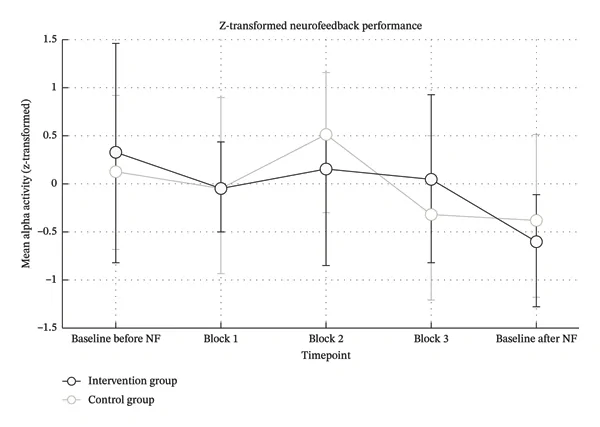
Z‐transformed alpha power across neurofeedback (NF) blocks. The *x*‐axis represents the timepoints for the different measures in Session 2, while the *y*‐axis represents the z‐transformed mean alpha activity. The black line represents the intervention group; the gray line represents the control group. Each timepoint includes dots, which indicate alpha power, and bars, which represent standard deviation. Between each timepoint, a tendency line is made.

No significant changes in alpha activity were observed before and after neurofeedback training in Session 2. Resting alpha activity did not differ significantly between pretraining with a median at −0.31851 (range −1.12049–2.10564) and post‐training with a median at −0.46257 (range: −0.96131–2.12823) (*n* = 28, *p* = 0.395, *r* = 0.164). Similarly, alpha activity during movement showed no significant change from pretraining with a median at −0.22267 (range: −0.98546–3.23354) and post‐training with a median at −0.49351 (range: −93917 to 2.47917) (*n* = 28, *p* = 0.058, *r* = 0.365).

#### 3.2.1. Neurofeedback Strategies

A summary of the strategies used by the participants for modulating alpha activity is shown in Figure 4.

**FIGURE 4:**
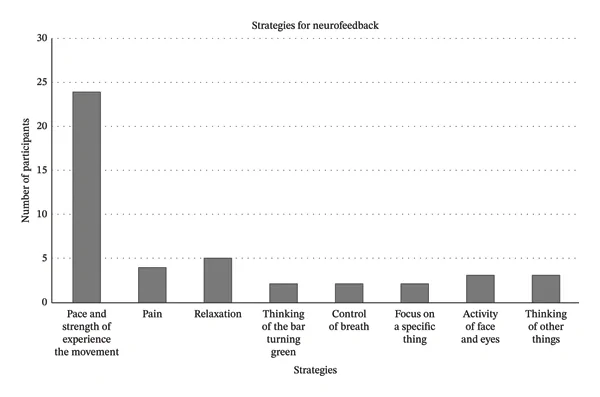
The distribution of participants using each type of strategies for neurofeedback. Here, the *x*‐axis represents the categories of neurofeedback strategies, while the *y*‐axis represents the number of participants who have used each type of strategy.

Multiple strategies per participant were allowed. Twenty‐four participants reported using strategies related to adjusting movement pace and strengths, which were equally distributed between the groups. To isolate cognitive strategies, changes in alpha activity during neurofeedback were reanalyzed for data of the combined groups, excluding these participants. No significant changes were observed in alpha activity across the neurofeedback blocks, even when focusing on no motor strategies (*n* = 12, *p* = 0.779, *W* = 0.042).

#### 3.2.2. Analysis of Success Rate

The average success rate across participants ranged between 22.2% and 58.3%, with a mean of 43.66%. None of the participants met the threshold for a successful neurofeedback training of 60% [30].

## 4. Discussion

This study investigated whether a single session of neurofeedback could modulate pain perception and alpha activity during acute MSK pain induced by DOMS. The study aimed to evaluate the effectiveness of the DOMS protocol in eliciting pain and to determine how DOMS‐induced pain affects alpha activity during movement and whether neurofeedback could successfully modulate alpha activity and reduce pain perception. The DOMS protocol effectively induced pain, as evidenced by significant increases in self‐reported pain rating. Additionally, DOMS resulted in a reduction of alpha activity during movement. However, one session of neurofeedback training did not result in significant modulation of alpha activity, nor did it lead to a measurable reduction in pain perception.

### 4.1. Comparison of Groups

No significant differences were observed between the intervention and control groups in age, gender, hand dominance, or alpha activity during movement in Session 1. However, a significant difference was found in the resting alpha activity at baseline. Given that participants were randomly assigned, this is likely due to random variation. Individual factors such as relaxation levels and cortical activity, known to influence alpha activity, may account for this variation [7]. This baseline difference between groups should be considered when interpreting group comparisons involving resting alpha activity, as it introduces potential confounding effects.

### 4.2. Effectiveness of DOMS Protocol

The DOMS protocol effectively induced pain, as evidenced by significant increases in NRS scores and reductions in alpha activity during movement between Sessions 1 and 2. These findings support the protocol’s validity for eliciting acute MSK pain. The significant increase in NRS 24–48 h after the induction of DOMS aligns with the literature showing that DOMS typically peaks within this time frame [22] This is consistent with findings by Dannecker et al. [27] who reported significant increases in the subjective experience of pain following the induction of DOMS [27]. Notably, Dannecker et al. [27] observed slightly higher pain ratings using the elbow flexor, potentially due to the predominance of Type II muscle fibers, which are more susceptible to DOMS [31]. Significant changes in PPT were not found, contrary to previous research indicating that DOMS increases VAS scores while decreasing PPT [32]. The discrepancy in findings may be due to the specific anatomic site chosen for PPT measurement at the exercised muscle, which may not have captured changes in muscle pressure sensitivity. This is supported by Weerakkody et al. [33], who demonstrated that DOMS affected the soreness heterogeneously even within the same muscle [33].

Resting alpha activity remained unchanged following DOMS induction, consistent with findings from Gervasio et al. [21], who reported no significant differences in resting alpha between healthy individuals and those with chronic pain [21]. Similarly, Moraes et al. [34] found no significant changes in resting alpha following exercise‐induced pain [34]. Overall, this indicates consistency in studies showing that MSK pain does not affect alpha activity during rest. Interestingly, while Gervasio et al. [21] found a significant increased alpha activity during movement in chronic pain patients [21], the current study observed a decrease following DOMS induction. This discrepancy may reflect differences in pain origin, chronic versus acute, and their respective neuroplastic effects. The literature on DOMS‐related alpha modulation remains limited. Plattner et al. [35] reported a significant increase in alpha activity over the somatosensory cortex during self‐paced biceps movement in participants with exercise‐induced pain, using a protocol similar to the present study. However, their findings contrast with the current results, possibly due to unreported differences in EEG processing or movement detection methods [35]. Overall, studies examine the impact of experimentally induced pain on alpha activity reporting mixed findings. A systematic review conducted by Mathew et al. [36] noted both increases and decreases in alpha following experimentally induced muscle pain using hypertonic injections, though the majority of the studies supported a decrease in alpha, as seen in the current study [36]. This variability highlights the need for further research on DOMS and its influence on cortical oscillations.

Theoretically, a decrease in alpha activity during movement is plausible, as alpha desynchronization in the somatosensory cortex is associated with movement planning and cognitive processing. Pain may intensify this desynchronization, potentially leading to further decrease in alpha power during movement [29]. However, pain perception is highly individual, and its effect on the alpha activity complex is incompletely understood.

### 4.3. Effectiveness of Neurofeedback

The neurofeedback protocol was overall ineffective; no significant changes in alpha activity were observed across the three neurofeedback blocks and the baseline measurement before and after neurofeedback. Both the intervention and control groups exhibited similar trends, with a general decrease in alpha activity over time, suggesting that group assignment did not influence outcomes.

One possible explanation for the lack of effect is the participants’ choice of strategy during neurofeedback. Several participants reported focusing on different motor aspects, such as adjusting the movement speed. However, previous literature suggests that movement‐related alpha activity is independent of movement duration and speed [29]. Thereby, using movement‐related strategies during neurofeedback may be suboptimal and ineffective. The emphasis on external movement rather than internal attention may have limited participants’ ability to engage with the neurofeedback task effectively. This interpretation is supported by the low success rate observed across participants.

When comparing, methodological differences between the current study and previous research may also account for the lack of neurofeedback effects. For instance, studies by Birch et al. [13] and Al‐Taleb et al. [16] reported positive outcomes of neurofeedback for both alpha modulation and pain reduction [13, 16]. However, both studies employed pre–post designs without control groups and are rated as moderate quality according to the National Heart, Lung, and Blood Institute quality assessment [17]. In contrast, the current study used a randomized design with an active control group, enhancing internal validity, but did not find any significant differences between the two groups. Other higher‐quality studies using controlled intervention designs have similarly reported limited effects. A study conducted by Rice et al. [18] found no significant differences between neurofeedback and sham groups, although both groups showed increases in alpha and reductions in pain [18]. Similarly, Ide‐Walters et al. [19], using a controlled design assessed as moderate quality, also reported a significant pain reduction in both a sham and an active neurofeedback group, with no significant group differences [19]. Overall, these findings indicate that the effects of neurofeedback might not be specific of the feedback itself and would need further investigation.

Participants’ characteristics may also play a role. The participants in this study were healthy individuals experiencing DOMS. Three of the four aforementioned studies, Birch et al. [13], Rice et al. [18], and Al‐Taleb et al. [16], involved chronic pain patients, who may find the neurofeedback more meaningful and therefore engage more deeply with the task [13, 16, 18]. Furthermore, this study used a single‐session design, in contrast to protocols with multisession protocols used in other studies [13, 18, 19]. For instance, Mrachacz‐Kersting et al. [20] reported reductions in alpha power and pain intensity after three sessions of neurofeedback over 3 days, although these results are based on only three participants and no control group limits its generalizability [20].

Overall, these findings suggest that effective neurofeedback may require repeated training to facilitate learning and neuroplastic changes. A single session may be insufficient to produce measurable effects, particularly in healthy individuals with transient pain.

### 4.4. Limitations

The neurofeedback effects could be affected by the limited signal processing it is possible to implement due to the real‐time updates of the interface. When examining the COBIDAS‐EEG guidelines by Organization for Human Brain Mapping (OHBM), it is recommended to implement an artifact removal in the processing pipeline [37]. However, many commonly used artifact correction methods, including independent component analysis and advanced source‐separation approaches, are computationally demanding and are typically performed offline. As neurofeedback required continuous real‐time updates of the visual feedback, these procedures were not implemented during the intervention. Although participant instructions, investigator monitoring, and electrode quality checks were used to minimize contamination, physiological artifacts may still have influenced the feedback signal. Muscle activity generally has spectral characteristics that differ from alpha‐band oscillations, suggesting that artifact regulation alone is unlikely to explain the observed effects. Nevertheless, residual contamination cannot be ruled out, and future studies may benefit from implementing online artifact correction methods to further improve signal specificity.

A limitation of this study design is the lack of a sham group. Both the intervention and control groups received active feedback, which makes it difficult to isolate placebo effects. Previous studies have shown no significant differences between sham and active neurofeedback for pain reduction [18, 19], and similar tendencies in both groups highlight the need for including a sham condition in future research.

A significant baseline difference in resting alpha activity was observed between groups prior to the intervention. Although no obvious methodological or participant‐related source of bias was identified, we cannot rule out that this imbalance resulted from random variation. Nevertheless, the difference introduces uncertainty regarding the interpretation of postintervention effects, as pre‐existing group differences may have influenced the magnitude or direction of subsequent changes. Consequently, the intervention effects on resting alpha activity should be interpreted with caution. Replication in larger samples and analyses specifically controlling for baseline values are needed to strengthen causal inference.

Moreover, participant instruction may also have affected the outcome. Many participants focused on movement regulation. Although previous work with an earlier version of the current system shows alpha modulation within one single session using similar instructions, that study only included four participants [23]. However, Autenrieth et al. [28] have found that individually selected strategies can have both beneficial and limiting effects. While providing an initial strategy may reduce variability and improve the earlier outcomes of the performance, it can also hinder learning by preventing participants from exploring alternative approaches [28]. Therefore, in a single‐session study design, more targeted guidance on modulating alpha activity in the somatosensory cortex may improve the neurofeedback outcomes.

Overall, a single neurofeedback session did not significantly affect brain activity or pain perception, likely due to methodological limitations such as short training duration, variability in participant strategies, together with limited EEG preprocessing. However, the observed alpha modulation after DOMS suggests that targeting neuroplasticity in MSK pain remains promising, and future studies should use optimized protocols to improve outcomes.

## Author Contributions

The study was designed by K.A.G., J.B.V., and S.G. The experiments were performed by K.A.G. and J.B.V. The data were analyzed by K.A.G. and J.B.V. The results were critically examined by all authors. K.A.G. and J.B.V. had a primary role in preparing the manuscript, which was edited by F.R.A. and S.G.

## Funding

No funding was received for this manuscript.

## Disclosure

All authors approved the final manuscript.

## Conflicts of Interest

The authors declare no conflicts of interest.

## Data Availability

The data that support the findings of this study are available from the corresponding author upon reasonable request.
